# Features and mechanisms of canonical and noncanonical genomic imprinting

**DOI:** 10.1101/gad.348422.121

**Published:** 2021-06

**Authors:** Courtney W. Hanna, Gavin Kelsey

**Affiliations:** 1 Epigenetics Programme, Babraham Institute, Cambridge CB22 3AT, United Kingdom;; 2 Centre for Trophoblast Research, University of Cambridge, Cambridge CB2 3EG, United Kingdom

**Keywords:** chromatin, DNA methylation, endogenous retroviruses, epigenetics, genomic imprinting

## Abstract

Genomic imprinting is the monoallelic expression of a gene based on parent of origin and is a consequence of differential epigenetic marking between the male and female germlines. Canonically, genomic imprinting is mediated by allelic DNA methylation. However, recently it has been shown that maternal H3K27me3 can result in DNA methylation-independent imprinting, termed “noncanonical imprinting.” In this review, we compare and contrast what is currently known about the underlying mechanisms, the role of endogenous retroviral elements, and the conservation of canonical and noncanonical genomic imprinting.

## Abstract (precis)

In this review, Hanna and Kelsey discuss what is known about the underlying mechanisms of establishing and maintaining allelic epigenetic marks, the role of endogenous retroviruses, and the potential conservation of canonical and noncanonical imprinting in mice and humans.

## Introduction to genomic imprinting

Genomic imprinting is the monoallelic expression of a gene based on parent of origin. Imprinted genes are essential for fetal and placental growth and development. It is hypothesized that imprinting arose in placental mammals due to the conflict between maternal and paternal genomes in the fetus to regulate maternal resources during and immediately after pregnancy, with maternal imprints repressing fetal growth while paternal imprints promote it (Moore and Haig 1991). To date, there are several examples of imprinted genes that fit this model, including key regulators of fetal growth such as the insulin growth factor 2 (IGF2) and its receptor IGF2R, which are reciprocally imprinted (DeChiara et al. 1990; Barlow et al. 1991; DeChiara et al. 1991; Weksberg et al. 1993).

Shortly after the discovery of the first imprinted genes, it was shown that imprinted gene expression was regulated by allelic epigenetic marks, in particular repressive DNA methylation, inherited from the parental germline (Bartolomei et al. 1993; Brandeis et al. 1993; Ferguson-Smith et al. 1993; Li et al. 1993). This canonical form of imprinting has since been characterized across mammals and is highly conserved at a number of imprinted gene clusters. Imprinted genes and their regulatory features have been most extensively characterized in the mouse and human genomes, and genome-wide screens have identified not only species-specific but also tissue-specific imprinting. It was recently demonstrated that several placental-specific imprinted genes in mice are in fact regulated by an alternative epigenetic mechanism, histone 3 lysine 27 trimethylation (H3K27me3) inherited from the maternal germline. This form of DNA methylation-independent imprinting has been termed “noncanonical” imprinting. Despite its recent discovery, noncanonical imprinting has already been shown to be distinct in its genomic characteristics and underlying mechanisms from canonical imprinting, opening up a new field of study. In this review, we discuss what is known about the underlying mechanisms of establishing and maintaining allelic epigenetic marks, the role of endogenous retroviruses, and the potential conservation of canonical and noncanonical imprinting in mice and humans (summarized in Table 1).

**Table 1.**
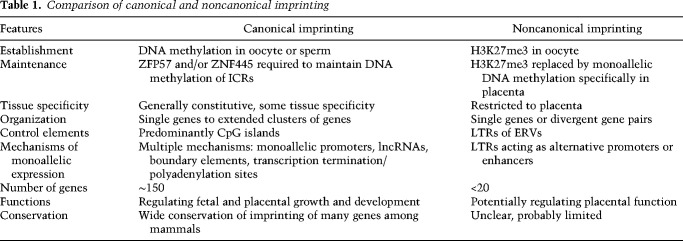
Comparison of canonical and noncanonical imprinting

## Mechanisms of canonical imprinting

### Gametic DNA methylation differences

The major driver of genomic imprinting has long been recognized as DNA methylation, specifically differences in methylation between the oocyte and sperm at imprinting control regions (ICRs). An ICR is the discrete genomic element that is necessary to orchestrate in *cis* the monoallelic expression of single or multiple imprinted genes within a domain (Spahn and Barlow 2003). ICRs coincide with germline differentially methylated regions (gDMRs), and most ICRs correspond to promoter CpG islands that acquire methylation in the female germline (Schulz et al. 2010).

**Figure 1.**
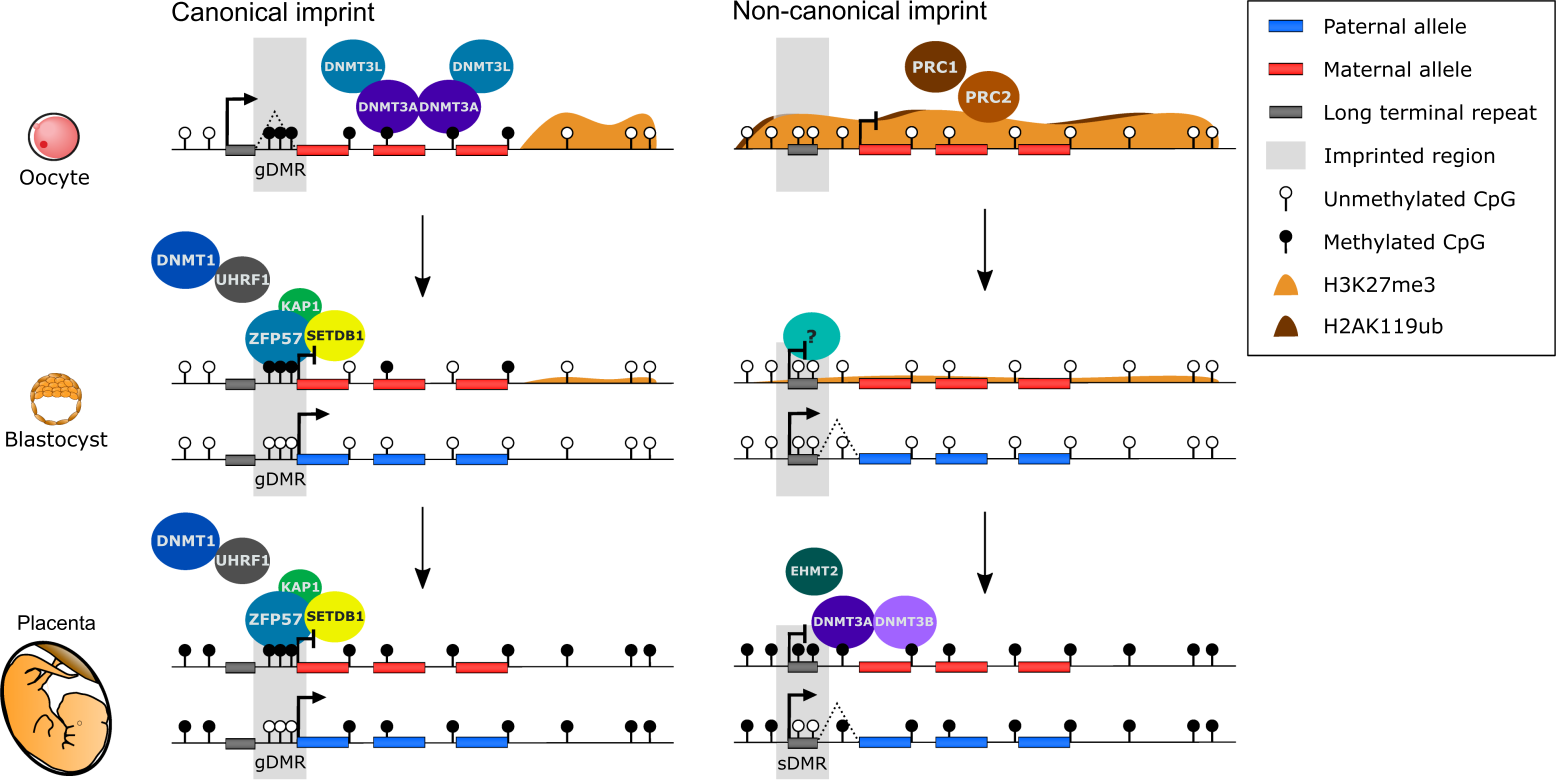
Mechanisms of establishment and maintenance of maternal canonical and noncanonical imprinting. (*Left*) Canonical imprinting: DNA methylation is targeted to transcribed gene bodies, including canonical imprinted gDMRs, in oogenesis by tetramers of DNMT3A and DNMT3L. There is widespread usage of long terminal repeats (LTRs) as alternative upstream promoters in the oocyte. In the preimplantation and postimplantation embryo, a complex of ZFP57 (or ZNF445), TRIM28 (KAP1), and H3K9 methyltransferase SETDB1 localizes to gDMRs recruiting DNMT1 to maintain DNA methylation on the maternal allele. In the postimplantation embryo, the gDMR is present in the fetus and placenta, enabling imprinted gene expression of a single gene or cluster of genes. (*Right*) Noncanonical imprinting: H3K27me3 is established by PRC2, which is in part dependent on PRC1 ubiquitination of H2AK119, across untranscribed regions in oogenesis, including noncanonically imprinted LTRs. In the preimplantation embryo, maternal H3K27me3 is progressively lost genome-wide, and whether an unknown factor is required to mark the maternal allele of noncanonically imprinted LTRs remains unclear. In the postimplantation embryo, noncanonical imprints become placental specific, acquiring DNA methylation on the maternal allele, creating secondary DMRs (sDMRs), in placental and extraembryonic cell types. In the fetus (not shown), the noncanonically imprinted LTRs become biallelically methylated. The acquisition of DNA methylation in postimplantation development at these domains is dependent on EHMT2 activity, through either the deposition of H3K9me2 or post-translational modification of proteins integral for de novo DNMT activity.

De novo methylation in germ cells requires the methyltransferase DNMT3A together with the catalytically inactive cofactor DNMT3L (Bourc'his et al. 2001; Hata et al. 2002; Bourc'his and Bestor 2004; Kaneda et al. 2004; Smallwood et al. 2011), which are recruited to an appropriate underlying histone modification landscape. In the oocyte, DNA methylation is almost exclusively restricted to transcribed gene bodies (Kobayashi et al. 2012). The widespread use of oocyte-specific alternative transcription start sites means that the majority of maternal ICRs are spanned by transcription (Fig. 1; Chotalia et al. 2009; Veselovska et al. 2015; Singh et al. 2017). The establishment of DNA methylation at maternal ICRs is a consequence of acquiring a permissive chromatin state for the recruitment of DNMTs. Loss of histone 3 lysine 4 dimethylation (H3K4me2) at intragenic CpG islands is catalyzed by the transcription-coupled lysine demethylase KDM1B (Ciccone et al. 2009; Stewart et al. 2015; Veselovska et al. 2015), and deposition of H3K36me2 and H3K36me3 over transcribed regions by the histone lysine methyltransferase SETD2 (Xu et al. 2019; Shirane et al. 2020). Conversely, sperm is highly methylated throughout much of the genome, a pattern that is conferred by DNMT3A and DNMT3L (Bourc'his and Bestor 2004; Kaneda et al. 2004), with the addition of DNMT3C in rodents (Barau et al. 2016). Unlike the oocyte, the deposition of DNA methylation in spermatogenesis is not dependent on H3K36me3, but rather H3K36me2, which shows broad genomic distribution through the activity of methyltransferase NSD1 (Shirane et al. 2020).

Thus, it appears that there is no mechanism of de novo methylation in the germline specifically targeted to imprinted loci per se. Rather, the distinctive dependence of the de novo DNMTs on H3K36 methylation in the oocyte and sperm results in dimorphic DNA methylation landscapes, providing the opportunity for imprinting to emerge at gDMRs. Consequently, locus-specific differences in gamete methylation between species is one mechanism that enables species-specific imprinting to arise (Brind'Amour et al. 2018).

### Postfertilization maintenance mechanisms

While distinct patterns of DNA methylation in the egg and sperm are the prerequisite for imprinting, gametic methylation differences are far more extensive than the number of imprinted loci; for example, there are ∼2000 CpG islands highly methylated in oocytes but not sperm (Kobayashi et al. 2012). Therefore, the maintenance of gamete-derived methylation in the embryo is critical in specifying the number of persistent gDMRs and, consequently, the number of imprinted loci. The discovery of the involvement of the zinc finger protein ZFP57 demonstrated that imprint maintenance relies on sequence-specific factors (Li et al. 2008; Mackay et al. 2008). ZFP57 is a member of the large family of Krüppel-associated box (KRAB)-containing zinc finger proteins (ZFPs) that provide DNA sequence binding specificity to the KRAB repressor complex. ZFP57 binds a CpG-containing hexanucleotide motif present in multiple copies in most ICRs (Quenneville et al. 2011; Strogantsev et al. 2015; Anvar et al. 2016) and, critically, binds the motif when the central CpG is methylated. ZFP57 recruits cofactors TRIM28 (KAP1) and SETDB1, targeting H3K9me3 to the locus, which in turn can be bound by the UHRF1 and DNMT1 complex following DNA replication, enabling maintenance methylation of the newly replicated DNA and the reinstatement of symmetric CpG methylation (Fig. 1; Sharif et al. 2007; Quenneville et al. 2011; Liu et al. 2013; Ming et al. 2020).

A second KRAB-ZFP, ZNF445, was recently identified as an alternative imprinting maintenance factor on the basis of its genomic binding profile in human and mouse embryonic stem cells, DNA methylation-dependent binding, and expression in human oocytes (Monteagudo-Sánchez et al. 2019; Takahashi et al. 2019). In mice, the two ZFPs both bind all imprinted DMRs and exhibit some functional redundancy, except for the *Peg10* DMR, raising the possibility that additional ZFPs may play a role in protecting imprints. Notably, based on differences in the severity of the phenotypes and gDMR methylation defects in zygotic mutants, ZFP57 appears to be the predominant methylation protective factor in mice (Takahashi et al. 2019). On the other hand, its greater evolutionary conservation and expression profile give more prominence to ZNF445 in maintenance of imprints in humans.

Although most germline differentially methylated CpG islands lose methylation during the phase of reprogramming in the preimplantation embryo (Smallwood et al. 2011; Kobayashi et al. 2012), some enjoy a transitory imprinted status before their methylation is erased or overwritten, a phenomenon referred to as transient imprinting (Proudhon et al. 2012). The significance of transient imprints is not fully understood; strikingly, however, their influence can outlast their very limited differential methylation status. In the case of the *Gpr1/Zdbf2* locus, monoallelic transcription of the long noncoding RNA (lncRNA) *Liz* from the *Gpr1* gDMR sets up persistent allelic differences in the embryo postimplantation (a secondary DMR [sDMR] and allele-specific enrichment of H3K27me3 upstream of the *Zdbf2* promoter), while the gDMR becomes biallelically methylated (Duffie et al. 2014). This early epigenetic programming event results in a long-term phenotypic effect: Mice deficient in *Liz* expression develop normally but do not activate *Zdbf2* in the brain postnatally and exhibit growth retardation (Greenberg et al. 2017).

### Imprinted long noncoding RNAs and *cis*-regulation of clusters

ICRs are incredibly potent genomic elements because they can specify the imprinted monoallelic expression of genes over tens of kilobases, or even megabases, of DNA. In the case of the *Airn/Igf2r* domain in the mouse placenta, imprinted expression extends to over a dozen genes spanning a genomic interval of >10 Mb (Andergassen et al. 2017). Much of the domain is also monoallelically enriched in H3K27me3 in extraembryonic tissues (Andergassen et al. 2019; Hanna et al. 2019). Deletion of the ICR (which is the promoter of the *Airn* lncRNA), or prevention of de novo methylation of the ICR in the oocyte, abolishes the imprinted status of the entire domain (Andergassen et al. 2017; Hanna et al. 2019). Just how an ICR enforces monoallelic transcription over such extended domains has been the focus of attention for many years. It is likely that a combination of mechanisms emanating from the ICR applies over such large domains, especially if there is sufficient evolutionary pressure to select imprinting of multiple genes; moreover, different mechanisms may have evolved at different imprinted clusters. For example, monoallelic silencing of *Igf2r* depends on interference of the promoter by *Airn* transcription, rather than the lncRNA itself (Latos et al. 2012). On the other hand, for genes in the domain not overlapped by *Airn* transcription, other mechanisms must operate. By extension of the model of promoter interference, one possibility is that monoallelic *Airn* transcription through essential placenta-specific enhancers represses the distal genes that depend on these enhancers. However, this model has been discounted by genetic experiments deleting the entire *Airn* transcribed region (Andergassen et al. 2019). This finding returns to the frame a long-established model that lncRNAs bind and recruit repressive chromatin modifiers, such as G9a (EHMT2) or polycomb repressor complexes (PRCs), to imprinted domains (Nagano et al. 2008; Terranova et al. 2008). For the megabase-scale imprinted domains, parallels with the lncRNA *Xist* and X-chromosome inactivation re-emerge (Khamlichi and Feil 2018). Molecular investigations in trophoblast and embryonic stem cells have demonstrated that 3D folding is essential to bring CpG islands within close proximity to ICRs at the *Airn* and *Kcnq1ot1* loci in *cis*, enabling PRCs to facilitate allelic silencing (Schertzer et al. 2019). Nevertheless, what features are critical for determining the extent of these imprinted domains and how exactly imprinted lncRNAs function remain to be fully elucidated.

## Noncanonical imprinting

### Discovery and properties

For many years, we have understood that DNA methylation is central to regulating imprinting; however, there have been examples of imprinted loci that appeared not to be controlled by DNA methylation, which compelled us to entertain alternative mechanisms of imprinting. For example, there were no detectable promoter DMRs at the placenta-specifically imprinted genes *Gab1* and *Sfmbt2*; in addition, their imprinting is retained even in conceptuses lacking oocyte-derived DNA methylation (Okae et al. 2012). An explanation for these anomalies has emerged with the discovery of a parallel mechanism of imprinting, which has been termed “noncanonical” imprinting.

Work that profiled DNase I-hypersensitive sites (DHSs) separately in isolated maternal and paternal pronuclei of mouse zygotes found that a subset of paternal-specific DHSs was not associated with known imprinted genes but with genes with paternal allele-biased expression (Inoue et al. 2017a). This was further evidenced from analysis of gynogenetic or androgenetic preimplantation embryos, as well as reciprocal hybrids. These genes do not map into regions of DNA methylation in oocytes, and their imprinting is maintained when oocytes are deprived of DNA methylation (Chen et al. 2019; Hanna et al. 2019). Critically, forced expression of the H3K27me3 demethylase KDM6B in zygotes abrogates their imprinted status (Inoue et al. 2017a). Genetic confirmation of the role of H3K27me3 has subsequently been obtained by conditional deletion of *Eed*, which encodes an essential component of the PRC2, in oocytes (Inoue et al. 2018).

An intriguing property of noncanonical imprinting is its tissue specificity. Although multiple genes with paternal allele bias in preimplantation embryos were identified, all (with the exception of *Slc38a4*) became biallelically expressed or repressed in the postimplantation epiblast (which gives rise to the embryonic lineages), while a subset retained imprinted expression in extraembryonic lineages and the placenta (Inoue et al. 2017a). This subset included *Gab1* and *Sfmbt2* previously identified as being independent of oocyte-derived methylation for their imprinting (Okae et al. 2012).

### Functions

Canonical imprinted genes participate in a range of developmental and physiological adaptations in mammals, notably in placental and fetal growth but also the central control of metabolism and some cognitive behaviors. It is too soon to conclude the functional domains of noncanonical imprinting, but the restriction of this form of imprinting to the placenta would implicate involvement in fetal growth control or placental endocrine adaptations to pregnancy. As a measure of its relative importance, global elimination of canonical imprinting by prevention of de novo DNA methylation in the female germline is incompatible with embryo development beyond mid gestation (Bourc'his et al. 2001). In contrast, abrogation of noncanonical imprinting by oocyte-specific ablation of *Eed* does allow survival to term but with reduced litter size, indicating embryonic losses (Inoue et al. 2018; Prokopuk et al. 2018).

A major role for oocyte-derived H3K27me3 has been identified in X-chromosome regulation. In rodents, the paternal X chromosome in female preimplantation embryos is silenced as a means of dosage compensation. This imprinted inactivation state persists in the extraembryonic lineages postimplantation, while in the embryo proper, both X chromosomes are reactivated before undergoing random X-chromosome inactivation. The epigenetic mark in the oocyte that suppresses the *Xist* locus to ensure activity of the maternal X chromosome in cleavage embryos has remained elusive, after reports that maternal DNA methylation was dispensable (Chiba et al. 2008). Recently, it was demonstrated that imprinted X-chromosome inactivation depended on oocyte H3K27me3 (Inoue et al. 2017b). Accordingly, both male and female embryos display aberrant inactivation of the maternal X chromosome upon oocyte-specific deletion of *Eed*, which could explain the excess of male embryo losses (Inoue et al. 2018). Because of this effect on X-chromosome dynamics, the global impact of perturbed noncanonical imprinting of autosomal genes is difficult to infer; in addition, there are discrepancies between studies. Oocyte-specific ablation of *Eed* is reported to result in reduced litter size and overgrowth of offspring but no sex ratio distortion (Prokopuk et al. 2018), or reduced litter size, deficit of males but ostensibly normal offspring (Inoue et al. 2018), while ablation of *Ezh2* in oocytes, which encodes the H3K27 methyltransferase, causes substantial growth retardation (Erhardt et al. 2003).

The importance of noncanonical imprinting has been demonstrated in somatic cell nuclear transfer (SCNT). In this procedure, nuclei from somatic cells are reprogrammed in the egg cytoplasm, but the process is inefficient and many cloned embryos fail owing, in part, to placental abnormalities. Whereas donor cells of embryonic or adult origin should have normal canonical imprints, they lack imprinting of noncanonical imprinted genes (Okae et al. 2014; Matoba et al. 2018). In an attempt to mitigate against this lack of noncanonical imprinting, SCNT was performed with donor cells carrying heterozygous deletions for each of the *Gab1*, *Sfmbt2*, and *Slc38a4* protein-coding genes to restore normal expression levels; however, none of the deletions prevented placental hyperplasia in clones. Strikingly, however, use of an allele that deleted the large miRNA cluster within an intron of *Sfmbt2* did substantially ameliorate the placental phenotype (Inoue et al. 2020).

Apart from *Sfmbt2*, noncanonical imprinted genes with significant phenotypic effects when ablated include *Slc38a4* (placental and fetal growth) (Matoba et al. 2019) and *Gab1* (placental labyrinth hypoplasia) (Itoh et al. 2000). However, the expression of either gene is not limited to extraembryonic tissues, and they have significant domains of imprinted or nonimprinted expression in other tissues, so it is difficult to conclude how much of the respective knockout phenotypes relate to their noncanonical imprinting. Thus, further work is needed to definitively demonstrate the importance of noncanonically imprinted genes in placental development.

### Establishment mechanism: targeting of H3K27me3 in the oocyte

In the oocyte, H3K27me3 is distributed in atypically broad domains and is anticorrelated with DNA methylation (Fig. 1; Zheng et al. 2016). Hence, H3K27me3 and any potential noncanonically imprinted domains are restricted to the untranscribed fraction of the genome. The mutual exclusivity of H3K27me3 and DNA methylation appears, at least in part, to be dependent on the establishment of H3K36me3 and DNA methylation across transcribed regions. Ablation of H3K36me3, and consequently DNA methylation, through the deletion of the H3K36me3 methyltransferase *Setd2* causes the patterning of H3K27me3 to become widespread (Xu et al. 2019). H3K27me3 and the self-interacting polycomb-associated domains (PADs)—a unique 3D organization of the oocyte genome—are established during oogenesis through the action of PRC2 and PRC1, respectively (Inoue et al. 2018; Du et al. 2020). Recent evidence further suggests that PRC2 activity is, at least in part, dependent on PRC1 ubiquitination of H2AK119, which is colocalized with the broad domains of H3K27me3 (Fig. 1; Mei et al. 2021). PRC2 enzymatic activity is tightly regulated throughout oogenesis through the action of the germline-specific PRC2 cofactor EZHIP, which represses deposition of H3K27me3 in the late stages of oogenesis (Ragazzini et al. 2019). In the absence of EZHIP, H3K27me3 is dramatically increased in mature oocytes, and female fertility is impaired.

Notably, PADs are lost in MII oocytes and reset postfertilization in the two-cell embryo, and it has been suggested that maternal H3K27me3 may be critical for this resetting (Du et al. 2020). However, as with H3K27me3, PADs are progressively lost during preimplantation development (Zheng et al. 2016; Du et al. 2020). At typical polycomb targets (e.g., *Hox* gene clusters), it has become evident that PRC1 activity at the two-cell stage is critical for later targeting of PRC2, but depletion of PRC1 in the zygote had no impact on noncanonical imprinting, suggesting it does not play a role in propagating maternal allelic silencing (Chen et al. 2021). These data support the notion that polycomb-independent mechanisms may be required for the maintenance of noncanonically imprinted domains past the embryonic cleavage stages.

### Maintenance mechanism: transition for H3K27me3 to DNA methylation control

What do we know about the mechanism of maintaining noncanonical imprints, and how does it differ from conventional DNA methylation-dependent imprinting? H3K27me3 is the mark inherited from oocytes that initially provides parental allele asymmetry by preventing establishment of DHSs and H3K4me3 on maternal alleles (Inoue et al. 2017a; Chen et al. 2019). However, H3K27me3 is reprogrammed in preimplantation embryos, resulting in very little genomic occupancy by the blastocyst stage (Zheng et al. 2016). DNA methylation is also reprogrammed globally in preimplantation embryos, but ICRs crucially are exempt from this loss. In contrast, noncanonical imprinted genes do not retain allelic H3K27me3 (Fig. 1), including in the extraembryonic tissues in which their imprinting persists (Chen et al. 2019; Hanna et al. 2019). Instead, almost all of these genes acquire sDMRs selectively in extraembryonic tissues (Fig. 1), such that their persistent imprinting is maintained by DNA methylation (Chen et al. 2019; Hanna et al. 2019). Recent evidence suggests that EHMT2 is critical in the establishment of sDMRs at noncanonically imprinted domains (Zeng et al. 2021), but it remains to be determined whether EHMT2-mediated H3K9me2 or histone-independent activities are required for DNA methylation establishment at these regions. Notably, the *Sfmbt2* locus is an exception in retaining maternal allele enrichment for H3K27me3 and lacking an sDMR (Andergassen et al. 2019; Chen et al. 2019). An important clue in understanding noncanonical imprinting regulation has been the identification of the long terminal repeats (LTRs) of endogenous retroviruses (ERVs) as the candidate regulatory elements (Hanna et al. 2019), as discussed in more detail below. Potentially, these LTRs could mediate both the activity of these elements at preimplantation stages and the placenta-specific expression, possibly via different transcriptional regulators, but their precise roles need to be experimentally determined.

## Endogenous retroviruses and genomic imprinting

ERVs are repetitive elements derived from retroviruses and comprise ∼10% of the mouse genome (Friedli and Trono 2015). Intact ERVs contain the retroviral genes necessary for viral replication, flanked by LTRs. However, the vast majority of ERVs in the genome have lost their viral genes through recombination between the complementary flanking LTRs and thus remain as “solo-LTRs” (Belshaw et al. 2007). LTRs have been frequently commandeered as *cis*-regulatory elements and significantly contribute to the gene regulatory landscape (Faulkner et al. 2009). LTR sequences can contain, or acquire through mutagenesis, sites for transcription factor binding, transcription initiation, splicing, and/or polyadenylation and thus can impact gene regulation in a multitude of ways (Thompson et al. 2016). Because of the mutagenic nature of retrotransposition events, intact ERVs are targeted for silencing by a rapidly evolving network of KRAB-ZFP proteins, which recruit H3K9me3 and DNA methylation (Matsui et al. 2010; Rowe et al. 2013; Ecco et al. 2016). However, solo LTRs often evade these silencing mechanisms and therefore have an increased likelihood of being co-opted into the gene regulatory landscape (Leung and Lorincz 2012). In particular, subclasses of solo LTRs appear to have benefitted from epigenetic reprogramming events in gametogenesis and early embryogenesis, acquiring essential roles in gene regulation during these developmental windows (Macfarlan et al. 2012; Chuong et al. 2013; Xie et al. 2013; Veselovska et al. 2015).

Shortly after the discovery of the first imprinted genes, Barlow (1993) proposed that genomic imprinting may have evolved as a host defense mechanism to silence viral genomic elements using DNA methylation. This hypothesis was based, in part, on work demonstrating that a transgene carrying an LTR acquired imprinting in gametogenesis, which persisted into the embryo (Chaillet et al. 1991). Since these early findings, there has been accumulating evidence that some aspects of genomic imprinting are linked to the silencing of LTRs and to transcriptional activity of solo-LTRs in the germline and placental trophoblast.

**Figure 2.**
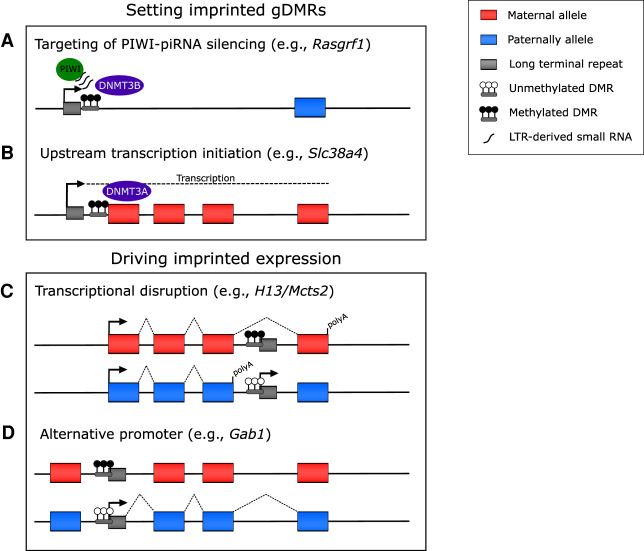
Role for LTRs in genomic imprinting. Endogenous retroviral LTRs have been demonstrated to be essential in both the setting of imprinted differentially methylated regions (DMRs) in the germline (*A*,*B*) and in driving imprinting gene expression at a number of loci (*C*,*D*). (*A*) In spermatogenesis, expression of LTR-derived small RNAs upstream of *Rasgrf1* is targeted by the piRNA-PIWI silencing pathway, which in turn recruits de novo methyltransferase DNMT3B to methylate the locus. (*B*) In oogenesis, the widespread occurrence of LTR-derived transcripts traversing canonical promoters results in their methylation via the recruitment of DNMT3A to sites of elongating transcription. (*C*) Within the intron of imprinted gene *H13*, an LTR-derived gene (*Mcts2*) harbors a maternal gDMR, resulting in imprinted expression of *Mcts2* from the paternal allele. The allelic transcriptional activity of *Mcts2* disrupts the transcriptional elongation of *H13,* causing its premature polyadenylation. (*D*) The allelic expression of several noncanonically imprinted genes is a consequence of LTRs acting as alternative promoters, forming chimeric transcripts with nearby genes.

The setting of imprinted DNA methylation in the male and female germlines has been linked to ERVs. There are three imprinted gDMRs established in spermatogenesis; however, only the *Rasgrf1* gDMR is attributable to an ERV. Expression of a solo-LTR (RMER4B) upstream of the *Rasgrf1* gene generates small RNAs that enable targeting of repressive PIWI-piRNA to the locus, resulting in its de novo methylation in sperm (Fig. 2A; Watanabe et al. 2011). Conversely, there is a widespread role for ERVs in the establishment of maternal imprinted gDMRs in oogenesis. As previously discussed, maternal gDMRs are predominantly located at CpG island promoters. In oogenesis, de novo DNA methylation is almost exclusively targeted to actively transcribed gene bodies (Kobayashi et al. 2012; Veselovska et al. 2015); thus, for imprinted maternal gDMRs to become methylated, they must be intragenic in the oocyte. This is achieved, in part, through the occurrence of transcriptionally active LTRs upstream of canonical promoters (Fig. 2B; Veselovska et al. 2015; Brind'Amour et al. 2018). Ablation of transcription across maternal gDMRs in the oocyte results in a failure of these regions to gain DNA methylation (Chotalia et al. 2009; Veselovska et al. 2015; Bogutz et al. 2019). As the majority of intergenic regions, and consequently LTRs, are unmethylated in the oocyte genome, it has yet to be explored why this specific subset of LTRs, mostly malRs and ERVKs, are active in oogenesis.

Several imprinted loci contain ERVs that facilitate their imprinting in the embryo. For example, the imprinted gene *H13* has an imprinted gDMR within its intron, which is a transcriptional start site for the retroviral-derived gene *Mcts2. Mcts2* is expressed from the paternal allele, and this transcription is proposed to interfere with the transcriptional elongation of paternal *H13*, resulting in its premature truncation and polyadenylation (Fig. 2C; Wood et al. 2008). At a number of noncanonically imprinted loci, LTR-initiated transcripts are spliced on to nearby protein-coding or noncoding RNA genes, resulting in imprinted chimeric transcripts (Fig. 2D; Hanna et al. 2019). Consistent with LTRs demonstrating tissue-specific activity, noncanonically imprinted LTRs are exclusively expressed in extraembryonic tissues, including the placenta and visceral endoderm (Hanna et al. 2019).

Imprinted gDMRs have also co-opted the ERV silencing machinery, KRAB-ZFPs, to enable the protection and maintenance of monoallelic DNA methylation during developmental reprogramming (Li et al. 2008; Pathak and Feil 2018), as previously discussed. While the vast majority of gDMRs do not contain an identifiable ERV, each (with the exception of one) contains motifs that are recognized by ZFP57 and/or ZNF445 (Quenneville et al. 2011; Takahashi et al. 2019). Notably, ZFP57 binds not only imprinted gDMRs but also a number of ERVs throughout the genome (Shi et al. 2019). Despite using a common KRAB-ZFP, the underlying mechanisms silencing imprinted gDMRs and ERVs appear to be distinct; in the absence of ZFP57, imprinted gDMRs become derepressed, while ERVs remain silenced (Shi et al. 2019). Beyond ZFP57 and ZNF445, there are also KRAB-ZFPs that act in a locus-specific manner. ZFP568 is essential for the establishment of DNA methylation at a sDMR at the placental-specific promoter of *Igf2* (Yang et al. 2017). Deletion of *Zfp568* results in up-regulation of *Igf2* and embryonic lethality, a phenotype that was partially rescued by deletion of *Igf2* (Yang et al. 2017). As the mechanisms regulating the establishment of sDMRs are investigated further, we may discover additional roles for KRAB-ZFPs in targeting allelic de novo DNA methylation in the postimplantation embryo.

Overall, ERVs have contributed to the evolution of genomic imprinting in mammals by several distinct mechanisms. The ability of LTRs to direct tissue-specific transcription, taking advantage of epigenetic programming events, has permitted (1) the expression of LTR-derived small RNAs that allow targeting of silencing piRNA-PIWI in spermatogenesis, (2) the targeting of DNA methylation to LTR-initiated transcription units in oocytes, and (3) the expression of noncanonical imprinted chimeric transcripts in the preimplantation embryo and placenta. Furthermore, the exploitation of the KRAB-ZFPs by gDMRs has enabled protection of their epigenetic state through early embryonic reprogramming events. With the advent of in vivo CRISPR technologies and ultra-low-input sequencing approaches, it is now possible to study and functionally test epigenetic reprogramming events in detail; these investigations may offer exciting new insights into the roles for ERVs in regulating genomic imprinting.

## Extent and conservation of imprinting

### Genome-wide surveys of imprinting

Since the early days of imprinting, there have been continual efforts to identify imprinted genes in a systematic manner, using the best current methods in species with useful available genetic resources or for which embryo manipulations were feasible. In the last decade, these efforts have been dominated by next-generation sequencing approaches, such as applying RNA-seq to reciprocal hybrid mouse crosses to quantify parental biases in expression using allelic variants. Early application of RNA-seq to the mouse brain resulted in the identification of >1000 genes exhibiting parent of origin allelic expression (Gregg et al. 2010), far higher than prevailing estimates of the number of imprinted genes. Such findings led to intense discussion about technical and bioinformatic errors in allelic RNA-seq data and the need for validation by independent methods, as well as debate about what threshold should be applied for calling an imprinted state from a parental allele expression bias (DeVeale et al. 2012; Kelsey and Bartolomei 2012). Subsequent studies have revealed an apparently complex repertoire of “nongenetic” allele-specific expression in the developing brain beyond conventional imprinting (Huang et al. 2017).

The issue of when parental-biased expression qualifies as imprinting is complicated by tissue complexity where cells with imprinted and nonimprinted expression for a given gene may exist side by side. Single-cell RNA-seq should help resolve this issue but may be limited by the problem of allele dropout. In recent single-cell analysis of the mouse cortex, consistent monoallelic/biased expression was found in all cells of the major cell types, at least for known imprinted genes (Laukoter et al. 2020); but this may differ for uncharacterized imprinted genes or genes at the extremities of imprinted domains. In the placenta, there is the additional issue of maternal tissue contamination, which has been a recurrent problem that probably led to an overestimate of the number of maternally expressed imprinted loci and dictates additional measures for verification (Okae et al. 2012; Andergassen et al. 2017). Nevertheless, it is possible that we are approaching a final listing of imprinted genes in mice: A recent RNA-seq survey of 34 tissues and developmental stages concluded with 93 high-confidence imprinted genes, and although this included 17 novel imprinted loci, they all belonged to characterized imprinted domains (Andergassen et al. 2017).

In human studies, an inevitable limitation is the inability to engineer crosses, necessitating other strategies to distinguish bona fide imprinted effects from allele-specific expression caused by *cis*-acting genetic variants. Recent surveys have mined large RNA-seq data sets from human tissues, supported by genotyped pedigrees or trios, to provide parent of origin information. Such studies have generally found known imprinted genes to be monoallelically expressed in multiple tissues, and provide a resource of novel candidates, many of which tend to exhibit tissue-restricted monoallelic expression (Babak et al. 2015; Baran et al. 2015). However, RNA-seq analysis based on short-read sequencing can have difficulties in reporting allelic biases of isoforms from complex transcription units, and tissue heterogeneity may still limit the ability to detect cell type-specific imprinting.

Genome-wide DNA methylation approaches to imprinted gene identification in humans have exploited abnormalities in imprinting, such as hydatidiform mole, uniparental disomies, or triploid placenta samples (Hanna et al. 2016; Sanchez-Delgado et al. 2016) or allelic variants (Hamada et al. 2016). A recent study took advantage of the unique genetic resource of the Icelandic population by conducting whole-genome bisulfite sequencing on peripheral blood DNA from 285 individuals for which parent of origin phased haplotypes, as well as RNA-seq data, were available (Zink et al. 2018). Such screens provide the basis for a comprehensive account of imprinting in humans, reveal the imprinting landscape in the human genome with a resolution previously only obtained in mice, and identify cases of polymorphic imprinting.

### LTRs and imprinting in humans

Many canonically imprinted gene clusters are conserved between mice and humans in both epigenetic regulation and synteny. This conservation extends to the role of LTR-derived transcripts in targeting DNA methylation in the oocyte and the requirement for ZFP57/ZNF445 in maintaining imprinted DNA methylation throughout early embryonic development. Nevertheless, some key differences have already been observed in the mechanisms of LTR-associated imprinting in humans, and the extent to which noncanonical imprinting may act on LTRs expressed in the human placenta has yet to be explored.

The genome of the human oocyte has more than twice as many methylated regions as the mouse oocyte, yet both exhibit DNA methylation largely restricted to transcribed domains (Okae et al. 2014; Hanna et al. 2018). LTRs contribute significantly in forming this transcriptional landscape, with >15% of all transcripts in the mouse oocyte initiating from an LTR (Veselovska et al. 2015). While LTRs contribute to a smaller proportion of the human oocyte transcriptome (Brind'Amour et al. 2018), LTR-initiated transcription mediates at least 15% of human-specific maternal gDMRs (Bogutz et al. 2019), demonstrating that LTR transcription in oocytes is a key player in the evolution of genomic imprinting.

A notable difference between mice and humans is the timing and necessity for KRAB-ZFPs in protecting imprinted gDMRs. Unlike in mice, *ZFP57* is not expressed in the human oocyte, meaning that the necessity for ZFP57 to protect imprinted DNA methylation must be later in development (Okae et al. 2014). Notably, *ZFP57* is also not expressed in bovine or porcine oocytes (Ivanova et al. 2020). Furthermore, mutations in *ZFP57* result in incomplete loss of imprinting (Bak et al. 2016), which may in part be explained by a predominant role for other ZFPs, such as ZNF445. ZNF445 appears to be complementary to ZFP57 in protecting human imprinted gDMRs and is highly expressed in human oocytes (Takahashi et al. 2019). However, it should also be noted that in the preimplantation human embryo, maternally inherited DNA methylation is not passively lost nearly to the extent it is in mice (Okae et al. 2014). Intriguingly, this persistence of inherited maternal DNA methylation appears particularly to affect the placenta, which exhibits hundreds of genomic loci with a maternal bias in DNA methylation (Hamada et al. 2016; Hanna et al. 2016; Sanchez-Delgado et al. 2016). Whether these placental-specific imprinted DMRs contain ZFP57/ZNF445 binding sites is currently unknown, but their polymorphic imprinting in the human population (Hanna et al. 2016; Sanchez-Delgado et al. 2016) suggests that, if present, there may be sequence variants in associated motifs.

### Recurrent evolution of noncanonical imprinted domains?

Given the evidence that noncanonical imprinting may be regulated by LTRs, and especially because the elements implicated in mice are rodent-specific, there is little expectation that the same genes will be imprinted in different mammalian lineages, although the process may be conserved across placental mammals. Consistent with this prediction, *GAB1* and *SFMBT2* are not imprinted in the human placenta (Okae et al. 2012); moreover, *GAB1* is not within a domain of H3K27me3 in human oocytes (Xia et al. 2019). *Slc38a4* is an interesting case because, in mice, the gene seems to combine canonical and noncanonical modes of imprinting. The promoter of *Slc38a4* contains a conventional maternal gDMR that depends on transcription from upstream MaLR elements in oocytes (Bogutz et al. 2019). Notably, the maintenance of allelic DNA methylation at the *Slc38a4* gDMR is uniquely dependent on H3K9 dimethyltransferase EHMT2 in the embryo (Auclair et al. 2016). Furthermore, *Slc38a4* is subject to noncanonical imprinted regulation in the placenta (Okae et al. 2012; Inoue et al. 2017a), possibly from upstream LTRs acting as enhancers that are within an H3K27me3-marked domain in oocytes (Hanna et al. 2019). *SLC38A4* is not imprinted in the human placenta (G Kelsey, unpubl.) but is reported to be imprinted in porcine placenta and polymorphically so in bovine placenta (Bischoff et al. 2009; Xu et al. 2018). The mechanism of imprinting in these species is not known. These observations suggest a selective pressure to imprint this gene and possibly independent evolutionary events. *Slc38a4* encodes the system A neutral amino acid transporter SNAT4, and mice lacking *Slc38a4* expression exhibit placental and fetal growth restriction (Matoba et al. 2019). The situation for the gene *Smoc1* is ambiguous: It is noncanonically imprinted in mice, and although imprinting of *SMOC1* has been described in human fibroblasts, the imprinted allele is opposite to that of mice (Santoni et al. 2017).

### Noncanonical imprinting: conserved or not?

An approach to evaluate the existence of H3K27me3-determined noncanonical imprinting in humans has been to examine the genomic distribution of H3K27me3 in human gametes or preimplantation embryos. Two studies have done this, but come to different conclusions. By performing CUT&RUN for H3K27me3 in human morulae, combined with whole-genome or exome sequencing of donor material to identify allelic variants, Zhang et al. (2019) identified regions of preferential maternal allele enrichment for H3K27me3. Furthermore, RNA-seq analysis identified genes with paternal allele-biased expression unlinked to sites of oocyte DNA methylation and therefore unlikely to be conventional imprinted genes. For one of these candidates, evidence of allelically enriched H3K27me3 was obtained. This could represent the type of transient imprinting observed in mouse preimplantation embryos, and it will be important to determine whether allelically biased expression persists in the placenta, as in noncanonical imprinting in mice. In contrast, in their survey of histone modifications in human oocytes and cleavage embryos, Xia et al. (2019) report that domains of H3K27me3 in oocytes are rapidly lost after fertilization and absent in the eight-cell stage, including at most of the above candidate genes, leading them to conclude that noncanonical imprinting is unlikely to exist in humans. Consistent with the absence of imprinted X-inactivation in humans (Migeon and Do 1979; Looijenga et al. 1999; Moreira de Mello et al. 2010), the *XIST* locus is devoid of H3K27me3 in preimplantation embryos (Xia et al. 2019).

**Figure 3.**
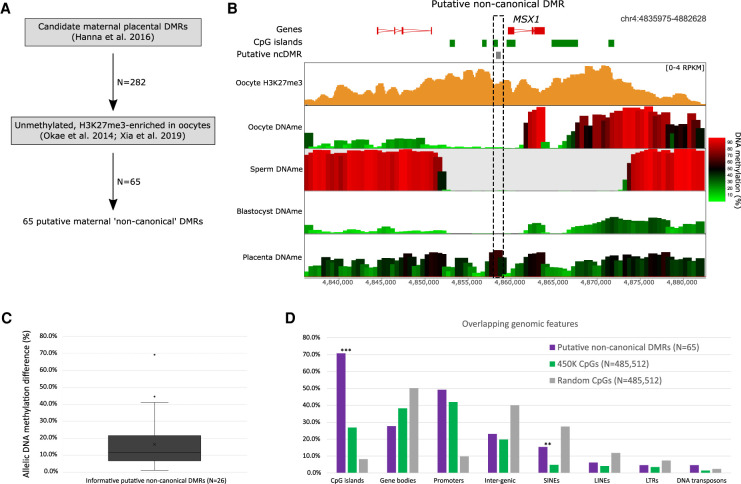
Identification of putative noncanonical imprinted DMRs in the human placenta. Using the epigenetic patterning characteristic of noncanonical DMRs in mice, we investigated publicly available data to identify putative maternal “noncanonical” DMRs in humans. (*A*) Previously reported candidate maternal DMRs from placenta were evaluated for oocyte DNA methylation and H3K27me3, selecting those domains that were unmethylated and enriched for H3K27me3 in oocytes (*N* = 65). (*B*) Screenshot of putative noncanonically imprinted DMR upstream of the *MSX1* gene*.* Enrichment for H3K27me3 in human oocytes is shown using running 500-bp windows, with a 100-bp step, quantitated as RPKM. DNA methylation in human oocytes, sperm, blastocyst, and first trimester placental trophoblast is shown using 1-kb running windows, with a 500-bp step. (*C*) The box plot shows the allelic difference in DNA methylation at informative putative noncanonical imprinted genes (*N* = 26). Informative DMRs were defined as those with at least three CpGs covered by at least two reads on each allele. (*D*) Overlapping genomic features were compared between putative noncanonical DMRs (*N* = 65) and CpGs on the Illumina 450K array (*N* = 485,512) using the χ^2^ statistic. *P*-value significance was adjusted for multiple comparisons using Bonferroni correction. (**) *P* < 0.001, (***) *P* < 0.0001. A random subset of genomic CpGs is shown in gray for context (*N* = 485,512).

Because noncanonical imprinted regions in mice transition from H3K27me3 in the oocyte to become imprinted sDMRs in placenta, we sought an alternative approach to identify potential noncanonically imprinted DMRs in humans. Taking advantage of candidate maternal placental DMRs identified previously using the Illumina HumanMethylation450 (450K) array (Hanna et al. 2016), we selected those that were unmethylated (<25%) and enriched for H3K27me3 (more than −0.75 log_2_RPKM corrected for DMR length) in human oocytes using publicly available data (Okae et al. 2014; Xia et al. 2019), resulting in 65 putative maternal “noncanonical” imprinted DMRs (Fig. 3A,B; Supplemental Table S1). We evaluated allelic DNA methylation by allelically mapping whole-genome bisulfite sequencing data from first trimester placental trophoblasts (Hamada et al. 2016), using the dbSNP common SNP annotation (http://www.ncbi.nlm.nih.gov/SNP) and the SNPsplit mapping program (Krueger and Andrews 2016). Allelic analyses confirmed a >10% allelic difference in DNA methylation at 16/26 of informative DMRs (Fig. 3C), although parent of origin was not assessed (Supplemental Table S1). Together, these data provide preliminary evidence that noncanonical imprinting may also exist in the human genome, while further work will be needed to validate candidate loci and demonstrate whether these DMRs regulate allelic expression. Notably, the putative noncanonical imprinted DMRs, compared with the Illumina 450K array probes, were significantly enriched for CpG islands and SINEs (Fig. 3D), rather than LTRs as in mice. This suggests that while the mechanism may be conserved between species, the underlying regulatory features are likely not. This difference may reflect the dissimilarities in the prevalence of repetitive elements between the mouse and human genomes (Thomas et al. 2003). However, it is important to highlight that repetitive elements in general are underrepresented on the Illumina 450K array (Fig. 3D); therefore, it is likely that loci have been missed by this approach.

The pursuit of comprehensively identifying human imprinted domains continues to present challenges, including the necessity for deep sequencing of genomics data sets, the scarcity of informative SNPs, obtaining parent of origin information for relevant SNPs, and the cellular heterogeneity of human samples, as previously discussed. The initial identification and characterization of noncanonical imprinting in mice emphasizes the value in using animal models to direct our approaches for investigating molecular and epigenetic phenomena in human development.

## Supplementary Material

Supplemental Material
