# Supplementary material for: Features and mechanisms of canonical and noncanonical genomic imprinting
Source: Genes Dev. 2021 Jun;35(11-12):821–34. doi: 10.1101/gad.348422.121 (PMC8168557; doi:10.1101/gad.348422.121)
Supplement: Supplemental Material [file supp_35_11-12_821__DC1.html]

Supplemental Material 

# Features and mechanisms of canonical and noncanonical genomic imprinting

## Supplemental Material

- Supplemental\_Table\_S1.xlsx
